# D-Dimer Is a Diagnostic Biomarker of Abdominal Aortic Aneurysm in Patients With Peripheral Artery Disease

**DOI:** 10.3389/fcvm.2022.890228

**Published:** 2022-06-03

**Authors:** Huoying Cai, Baihong Pan, Jie Xu, Shuai Liu, Lei Wang, Kemin Wu, Pu Yang, Jianhua Huang, Wei Wang

**Affiliations:** ^1^Department of General and Vascular Surgery, Xiangya Hospital, Central South University, Changsha, China; ^2^Department of Epidemiology and Health Statistics, School of Public Health, Central South University, Changsha, China; ^3^National Clinical Research Center for Geriatric Disorders, Xiangya Hospital, Central South University, Changsha, China

**Keywords:** abdominal aortic aneurysm, peripheral artery disease, D-dimer, fibrinogen, platelet count

## Abstract

**Background:**

Etiology and risk factors of peripheral artery disease (PAD) include age, smoking, and hypertension, etc. , which are shared by an abdominal aortic aneurysm (AAA). Concomitance with AAA in patients with PAD is not rare but is easily overlooked in the clinical situation, though management strategies are altered totally. This study aims to investigate diagnostic biomarkers for the prediction of AAA in patients with PAD.

**Methods:**

A total of 684 patients diagnosed with AAA and/or PAD were enrolled and analyzed retrospectively. Each patient with PAD and AAA was gender and age-matched. Demographic data, medical history, and serum laboratory test profiles were obtained. Statistical analysis was performed to determine diagnostic biomarkers of AAA in patients with PAD.

**Results:**

Firstly, 320 patients with PAD-only and 320 patients with AAA-only were compared. Levels of bilirubin and D-Dimer were decreased, while the incidence of diabetes mellitus, levels of fibrinogen, and platelet count were increased significantly in patients with PAD-only compared with those in patients with AAA-only (*P* < 0.001). Next, 364 patients with PAD (44 patients with AAA) and 364 patients with AAA (44 patients with PAD) were compared. Multivariate logistic regression analysis confirmed the differential distribution of bilirubin, D-dimer, fibrinogen, and platelet count between patients with AAA and patients with PAD (*P* < 0.05). Receiver operator curves (ROC) showed that the area under the curve (AUC) of total bilirubin, direct bilirubin, D-dimer, fibrinogen, and platelet count was 0.6113, 0.5849, 0.7034, 0.6473, and 0.6785, respectively. Finally, to further validate the predictive efficacy of mentioned markers, a multivariable logistics regression analysis was performed between the PAD only group and the PAD with AAA group. The results suggested increased levels of D-dimer in the PAD with AAA group compared to the PAD only group (OR: 2.630, 95% CI:1.639–4.221; *P* < 0.001). In particular, the Youden index suggested that the cut-off value of D-dimer for predicting AAA in patients with PAD was 0.675 mg/L with a sensitivity of 76.9% and a specificity of 84.9% (AUC = 0.8673; 95% CI, 0.8106–0.9240, *P* < 0.001). In all 364 patients with PAD, 41.46% patients were diagnosed AAA when D-dimer is >0.675 mg/L, while only 3.55% patients were diagnosed AAA when D-dimer ≤ 0.675 mg/L.

**Conclusions:**

PAD and AAA exert different clinical and serum profiles; D-dimer (>0.675 mg/L) is a reliable biomarker for the prediction of AAA in patients with PAD.

## Introduction

Abdominal aortic aneurysm (AAA) is defined as the dilation of the abdominal aortic artery (≥30 mm, ≥50% normal size) (1, 2). With the aging of the population, the incidence of AAA has been rising significantly, which has become a serious problem threatening health conditions (3, 4). In particular, mortality of ruptured AAA ~90% if untreated and 40% if treated (5, 6). Early diagnosis and careful monitoring are key to better managing the issue.

Peripheral arterial disease (PAD) is a chronic atherosclerotic disease. PAD leads to chronic ischemia of the lower extremity, manifesting as intermittent claudication or critical limb ischemia. PAD occurs mainly in elderly male patients (7). The prevalence of PAD was up to 10% around the world and is associated with increased mortality and morbidity (8).

Risk factors of PAD include age, gender (male), smoking, hypertension, abnormal blood lipid (8–14), and others, which are shared by AAA. The etiology of these two diseases is also atherosclerosis (15, 16), meaning it is clinically possible that patients with the mentioned risk factors can have PAD and AAA simultaneously (12.1% in the present study), which is easily overlooked by clinicians given that AAA is mostly asymptomatic. However, therapeutic options may vary among those patients. For example, patients with PAD may need first to receive surgical repair of AAA if the diameter is ≥55 mm or careful monitoring of the aorta if the diameter is ≤ 55 mm.

Therefore, markers to predict AAA in patients with PAD are needed to facilitate health care providers to better screen out AAA in patients with PAD. To date, relatively few studies have specifically investigated the potential value of a blood marker-based approach.

### Aim

The aim of this study is to: (1) investigate the similarity and differentia of risk factors of the two diseases, and (2) to explore serum-based diagnostic biomarkers to predict AAA in patients with PAD.

## Materials and Methods

### Enrollment of Study Objects

**Figure 1:**
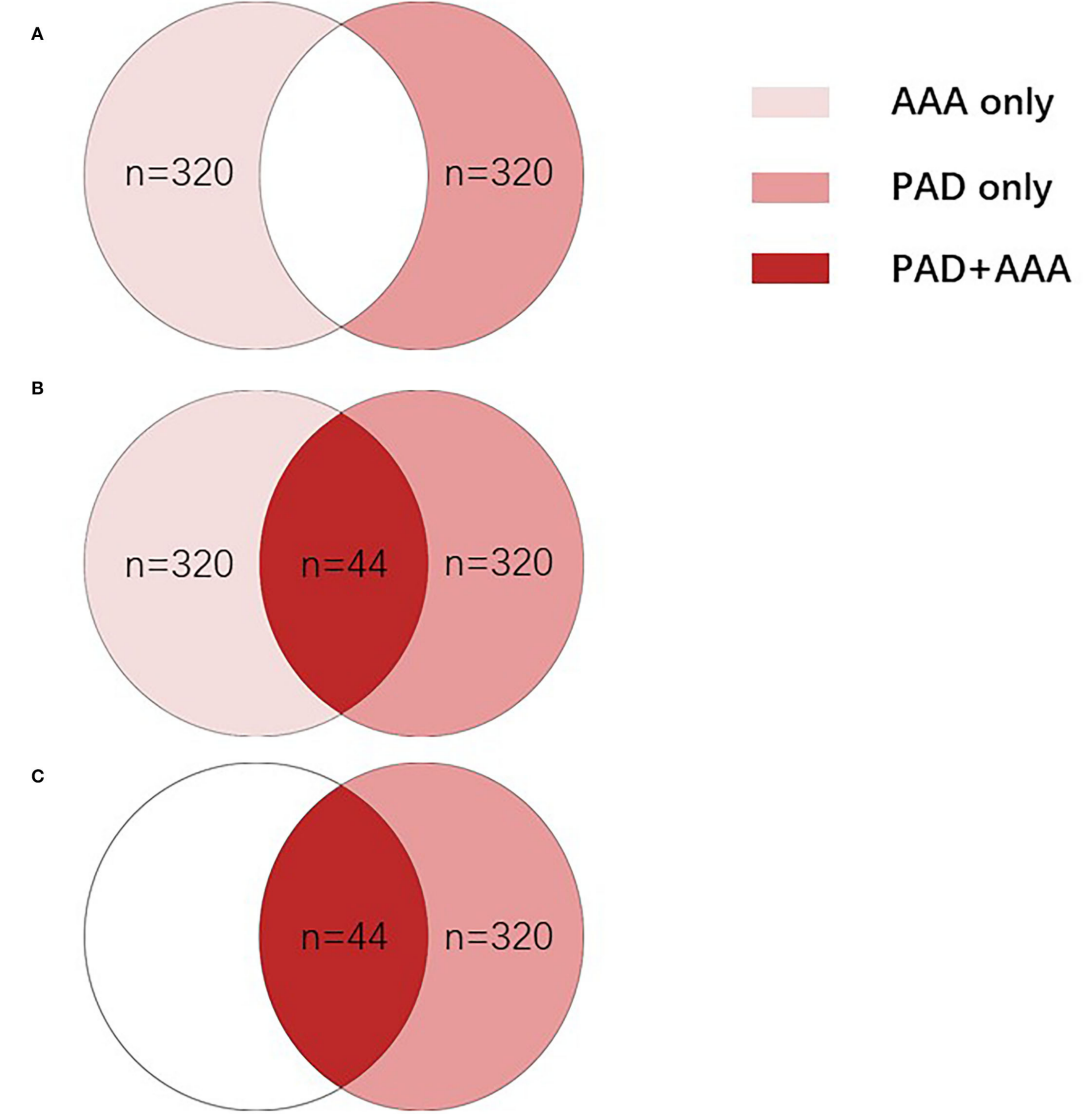
All patients were divided into different groups based on diagnosis. **(A)** To compare the difference between the PAD group and AAA group, AAA only group (*n* = 320), in which patients were diagnosed with AAA without PAD, and the PAD only group (*n* = 320), in which patients were diagnosed with PAD without AAA were enrolled. **(B)** To evaluate whether different indicators can be used to distinguish patients with PAD and patients with AAA, all 684 patients were divided into two groups: the AAA group (*n* = 364), in which patients were diagnosed with AAA only (*n* = 320) or both AAA and PAD (*n* = 44); and the PAD group (*n* = 364), in which patients were diagnosed with PAD only (*n* = 320) or both PAD and AAA (*n* = 44). **(C)** To evaluate whether d-dimer can clinically distinguish patients with PAD with and without AAA, 364 patients were enrolled, including the PAD only group (*n* = 320), in which patients were diagnosed with AAA without PAD and the PAD + AAA group (*n* = 44), in which patients were diagnosed with both AAA and PAD. AAA, abdominal aortic aneurysm; PAD, peripheral arterial disease.

Six hundred and eighty-four patients who were diagnosed with AAA and/or PAD at the department of vascular surgery, Xiangya Hospital, Central South University from January 2010 to May 2019 were enrolled in this study. Patients can be divided into different groups based on diagnosis: including the AAA only group (*n* = 320), in which patients were diagnosed with AAA, but not with PAD; the PAD only group (*n* = 320); in which patients were diagnosed with PAD but not with AAA; and the PAD + AAA group (*n* = 44), in which patients were diagnosed with both AAA and PAD (Figure 1). All procedures were approved by the Institute Review Board of Xiangya Hospital, Central South University (IRB No. 201905267). Patients were informed, and consent forms were obtained for research purposes.

Including criteria for AAA: (1) Age >40 years old; (2) AAA was confirmed by CTA or MRA.

The exclusion criteria are as follows: (1) infectious AAA; (2) secondary AAA with certain causes (such as injury); (3) severe mental illness leading to incapability of cooperation; (4) severe infectious or autoimmune diseases; (5) pregnancy or malignant tumor; and (6) thrombotic diseases such as venous thromboembolism.

The inclusion criteria for PAD were: (1) Age > 40 years; (2) High-risk factors such as smoking, diabetes, hypertension, hyperlipidemia, and others; (3) Symptoms of the ischemic lower extremity; (4) The pulse of the distal arteries of the ischemic limb is weakened or disappeared; (5) Ankle-brachial index (ABI) of ≤ 0.9; (6) Artery stenosis or occlusion were confirmed by CTA or ultrasound. The clinical diagnosis of PAD can be made if four of the above diagnostic criteria were met.

The exclusion criteria of PAD are as follows: (1) severe infectious or connective tissue diseases; (2) pregnancy or malignant tumor; (3) severe mental illness leading to the incapability of cooperation; and (4) thrombotic diseases such as venous or arterial thromboembolism.

### Information Collection

Demographic characteristics, history, and personal history were recorded. Peripheral blood was collected at the time of administration. Specifically, levels of D-dimer were obtained at the time of admission by enzyme-linked immunosorbent assay. Laboratory tests were performed and data were collected.

### Statistical Analysis

Kolmogorov-Smirnov test was used to determine whether quantitative data were normally distributed. Quantitative data are shown as a mean ± standard deviation (SD) for normally distributed variables or median (interquartile range) for non-normally distributed variables. Qualitative data are presented as relative frequencies. Chi-square test or Fisher exact test was performed among qualitative data, and Student's *t*-test or Mann Whitney *U*-test was conducted among quantitative data. Multivariate logistic regression analysis was utilized to determine the differential distribution of biomarkers. Receiver operating characteristics (ROC) curves and the area under the curve (AUC) were performed to further assess the diagnostic efficacy of biomarkers. All statistical analyses were performed using the Statistical Product and Service Solutions (SPSS) 22.0 statistical software package (IBM Analytics, Armonk, NY, USA).

## Results

### Incidence of Diabetes Mellitus and Levels of D-Dimer, Fibrinogen, and Platelet Count Are Differentially Distributed Among Patients With PAD and AAA

Demographic and medical history were recorded. To screen the potential differentially distributed parameters in patients with AAA and patients with PAD, statistical analysis was performed in AAA only group (*n* = 320) and PAD only group (*n* = 320). As shown in Table 1, the incidence of diabetes mellitus decreased significantly in AAA only group compared to PAD only group (11.25 vs. 31.56%, *P* < 0.01). No obvious difference in age, gender, hypertension, dyslipidemia, coronary heart disease, cerebral infarction, smoking, or drinking was founded between AAA only group and PAD only group (*P* > 0.05).

**Table 1:** Demographic and Clinical Characteristics of patients in AAA only and PAD only group.

**Parameter**	**AAA only *n* = 320**	**PAD only *n* = 320**	***P*-value**
Age (years)	67.66 ± 7.96	68.23 ± 7.95	n.s.
Male, *n* (%)	266 (83.1%)	266 (83.1%)	n.s.
Hypertension, *n* (%)	197 (62.56%)	197 (62.56%)	n.s.
Dyslipidemia, *n* (%)	109 (64.50%)	113 (63.66%)	n.s.
DM, *n* (%)	36 (11.25%)	101 (31.56%)	<0.01
CAD, *n* (%)	98 (30.52%)	87 (27.19%)	n.s.
CI, *n* (%)	30 (9.37%)	42 (13.12%)	n.s.
Smoking, *n* (%)	214 (66.87%)	224 (70.00%)	n.s.
Drinking, *n* (%)	114 (35.62%)	120 (37.5%)	n.s.

*Quantitative data are presented as mean ± standard deviation or median (interquartile) and are compared with the Student's t-test or Mann Whitney U-test. Qualitative data are presented as relative frequencies and are compared with Chi-square test or Fisher exact test. AAA, abdominal aortic aneurysm; PAD, peripheral artery disease; DM, diabetes mellitus; CAD, coronary artery disease; CI, cerebral infarction; n.s., not significant (p > 0. 05)*.

To screen the potential differentially distributed serum markers among patients with PAD and patients with AAA, peripheral blood samples were collected at the time of admission, and laboratory tests were performed (liver function, renal function, lipids, coagulation, and platelet count). As shown in Table 2, levels of total bilirubin, direct bilirubin, and D-dimer were increased, while levels of fibrinogen and platelet were decreased significantly in AAA only group when compared with those in PAD only group (*P* < 0.001). No obvious difference in TBA, BUN, SCR, uric acid, fasting glucose, low-density lipoprotein cholesterol, high-density lipoprotein cholesterol, triglyceride, total cholesterol, PT, or hemoglobin were found between AAA only group and PAD only group (*P* > 0.05). This suggests that patients with AAA may experience relatively high levels of bilirubin and D-dimer, while patients with PAD may show relatively high levels of fibrinogen and platelet.

**Table 2:** Blood Laboratory test profiles of patients in AAA only and PAD only group.

**Parameter**	**AAA only** ***n* = 320**	**PAD only** ***n* = 320**	***P-*value**
Total bilirubin (umol/L)	9.80 (7.12/13.10)	7.90 (5.50/10.57)	<0.001
Direct bilirubin (umol/L)	3.90 (2.90/5.40)	3.35 (2.40/4.40)	<0.001
TBA (umol/L)	4.20 (2.40/6.82)	3.95 (2.50/7.07)	0.936
BUN (mmol/L)	5.64 (4.56/7.18)	5.58 (4.28/7.45)	0.327
SCR (umol/L)	94.00 (81.00/112.90)	91.00 (77. 25/110.75)	0.077
Uric acid (umol/L)*	349.43 ± 104.15	347.09 ± 102.80	0.775
Fasting glucose(mmol/L)	5.17 (4.70/5.97)	5.22 (4.51/6.08)	0.852
TG (mmol/L)	1.35 (0. 93/1.91)	1.50 (1.11/2.44)	0.463
TC (mmol/L)	4.59 (3.83/5.44)	4.52 (3.73/5.19)	0.090
HDL-C(mmol/L)*	1.10 ± 0.32	1.19 ± 0.25	0.501
LDL-C (mmol/L)	2.92 (2.33/3.54)	3.70 (2.85/4.35)	0.118
PT(s)	13.00 (12.50/13.80)	13.00 (12.40/13.80)	0.381
Fibrinogen (g/L)	3.46 (2.73/4.37)	4.45 (4.02/5.75)	<0.001
D-dimer (mg/L)	0.80 (0.45/1.52)	0.280 (0.18/0.46)	<0.001
Hemoglobin(g/L)*	123.80 ± 18.77	123.70 ± 20.82	0.949
Platelet count, *10^9^/L	168.00 (138.25/213.50)	221.00 (168.00/294.00)	<0.001

Data is presented as medians (interquartile range, Q1/Q3) or mean ± standard deviation and are compared with the Student's t-test (marked with *) or Mann Whitney U-test. AAA, abdominal aortic aneurysm; PAD, peripheral artery disease; TBA, total bile acid; BUN, blood urea nitrogen; SCR, serum creatinine; TG, triglyceride; TC, total cholesterol; HDL, high-density lipoprotein; LDL, low-density lipoprotein.

The absolute value of circulating parameters in Table 2 cannot fully reflect the health status of patients since whether those markers are in the normal range is more clinically relevant. Thus, patients in the AAA only group and PAD only group are sub-grouped into a normal or abnormal group (Table 3). Statistical analysis was carried out to validate the association of variables with AAA and PAD. As shown in Table 3, patients with AAA-only manifested a higher possibility of having an abnormal status of D-dimer, fibrinogen, and platelet count, and a lower possibility of diabetes mellitus when compared with patients with PAD-only (*P* < 0.001). No obvious difference in the abnormal status of total bilirubin and direct bilirubin was identified between AAA only group and PAD only group (*P* > 0.05), which is reasonable due to the median (Q1/Q3) value of total bilirubin and direct bilirubin in Table 2 are in the normal range (normal range for total bilirubin and is ≤ 17.1 and ≤ 6.8, respectively).

**Table 3:** Differential distribution of clinical and blood laboratory test parameters in AAA only and PAD only group.

**Parameters**	**Status**	**AAA only *n* (%)**	**PAD only *n* (%)**	***P*-value**	**OR (95% CI)**
Diabetes mellitus	Yes	36 (26.3)	101 (73.7)	<0.001	0.275 (0.181–0.418)
	No	284 (56.5)	219 (43.5)		
Total bilirubin	normal	33 (55.9)	26 (44.1)	>0.05	1.300 (0.758–2.229)
	*abnormal*	287 (49.4)	294 (50.6)		
Direct bilirubin	normal	34 (54.0)	29 (46.0)	>0.05	1.193 (0.708–2.010)
	*abnormal*	286 (49.6)	291 (50.4)		
D-dimer	*normal*	93 (26.9)	253 (73.1)	<0.001	9.217 (6.420–13.232)
	abnormal	227 (77.2)	67 (22.8)		
Fibrinogen	normal	107 (37.4)	179 (62.6)	<0.001	0.396 (0.287–0.545)
	*abnormal*	213 (60.2)	141 (39.8)		
Platelet count	normal	284 (48.1)	306 (51.9)	0.001	0.361 (0.191–0.683)
	*abnormal*	36 (72.0)	14 (28.0)		

*Data is compared with the Chi-square test or Fisher exact test. Normal range of total bilirubin, direct bilirubin, D-dimer, fibrinogen and platelet count, provided by the Department of Laboratory Medicine, is ≤ 17.1, ≤ 6.8, ≤ 0.5, ≤ 4, and ≥100, respectively. AAA, abdominal aortic aneurysm; PAD, peripheral artery disease; CI, confidence interval; OR, odds ratio*.

Together, the above results suggested that diabetes mellitus, D-dimer, fibrinogen, and platelet count were differentially distributed in patients with AAA when compared to patients with PAD.

### D-Dimer, Fibrinogen, and Platelet Count Can Be Used to Distinguish Between Patients With AAA and Patients With PAD

The AAA and PAD share common risk factors including atherosclerosis, age, gender (male), smoking and hypertension, and others. This makes it clinically realistic that a patient with PAD is concomitant with AAA, which is easily overlooked by clinicians, although management strategies are altered totally. To find out variants to distinguish between patients with AAA and patients with PAD, multivariable logistics regression analysis was performed in the AAA group (*n* = 364, Figure 1B) and PAD group (*n* = 364, Figure 1B). Results in Table 4 indicated that total bilirubin, direct bilirubin, D-dimer, fibrinogen, and platelet count could be used to distinguish between patients with AAA and patients with PAD (*P* < 0.05).

**Table 4:** Risk factors in AAA group and PAD group.

**Risk factors**	**OR (95% CI)**	***P-*value**
Total bilirubin (umol/L)	1.159 (1.038–1.295)	0.009
Direct bilirubin (umol/L)	0.778 (0.613–0.987)	0.038
Fibrinogen (g/L)	0.838 (0.726–0.968)	0.016
D-dimer (mg/L)	1.531 (1.244–1.885)	<0.001
Platelet count, 10^9^/L	0.995 (0.992–0.997)	<0.001

*Multivariable logistics regression analysis was performed in AAA group and PAD group. AAA, abdominal aortic aneurysm; PAD, peripheral arterial disease; CI, confidence interval; OR, odds ratio*.

**Figure 2:**
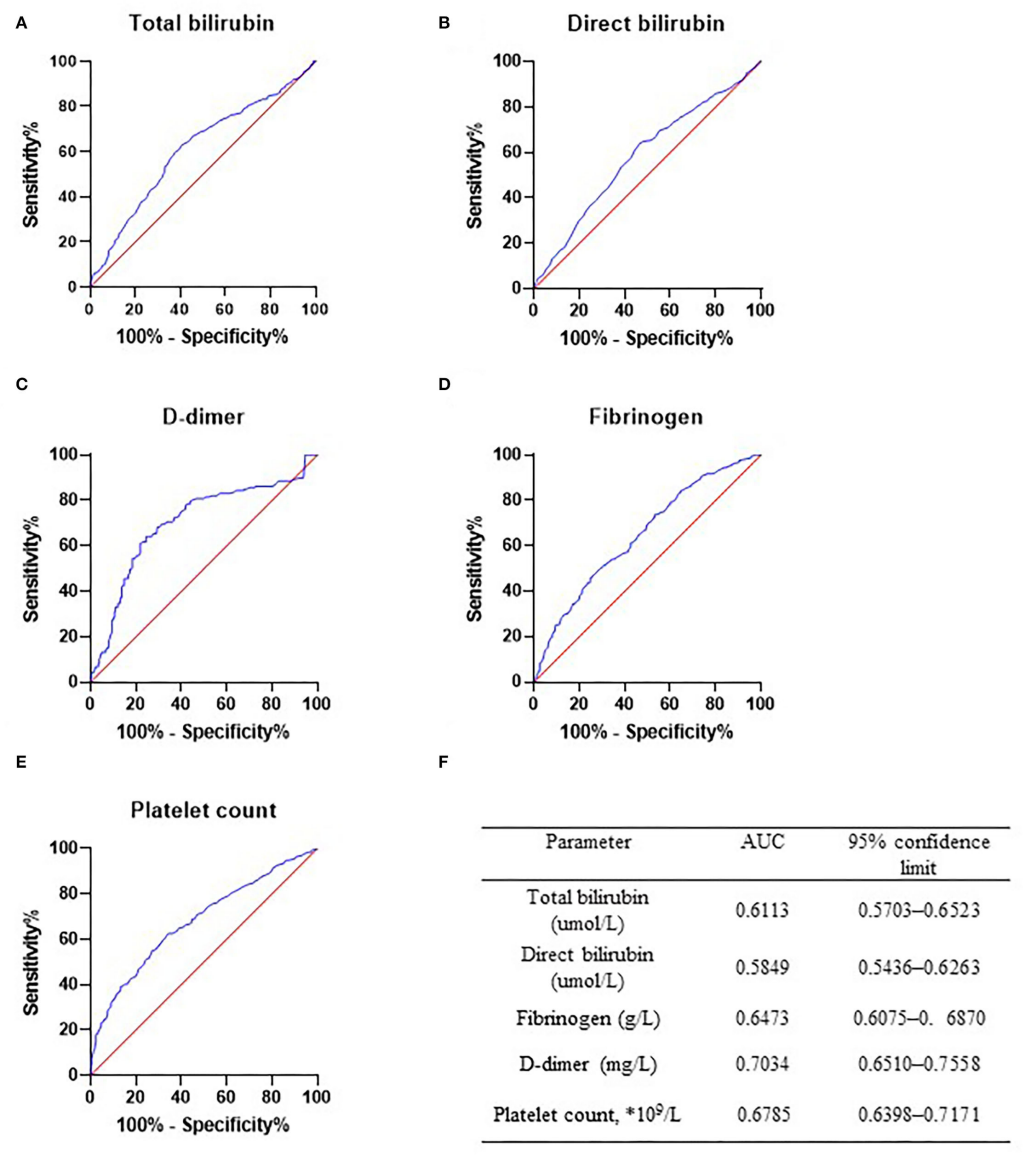
Receiver operator characteristic curve in abdominal aortic aneurysm (AAA) group and peripheral arterial disease (PAD) group. Receiver operator characteristic curve of total bilirubin **(A)**, direct bilirubin **(B)**, D-dimer **(C)**, fibrinogen **(D)**, and platelet count **(E)** was drawn in PAD only and AAA only patients. Diagnostic efficacy of these markers was determined. **(F)** Detailed information of ROC curves. AAA, abdominal aortic aneurysm; PAD, peripheral arterial disease.

To further evaluate the diagnostic efficacy of the mentioned markers, the receiver operating characteristic (ROC) curve of the five risk factors mentioned above was performed in the AAA group (*n* = 364, Figure 1B) and PAD group (*n* = 364, Figures 1B, 2). Figure 2F summarizes detailed information on ROC curves in Figure 2. As indicated in Figure 2F, D-dimer may be the most optimal biomarker for the distinction between patients with AAA and patients with PAD with the area under the curve (AUC) of 0.7034.

### D-Dimer Is a Reliable Diagnostic Biomarker of AAA in Patients With PAD Patients

As mentioned above, the main objective of this study was to find out ideal biomarkers for the prediction of AAA in patients with PAD. Total bilirubin, direct bilirubin, D-dimer, fibrinogen, and platelet may potentially be predictive biomarkers of AAA, though OR value or AUC was not that promising between patients with AAA (*n* = 364) and patients with PAD (*n* = 364). To further validate the efficacy of these biomarkers in a more clinically realistic condition, statistical analysis was performed again between the AAA + PAD group (*n* = 44) and the PAD group (*n* = 364).

As shown in Table 5, multivariate logistic regression analysis showed that the serum levels of D-dimer increased in AAA + PAD group compared with the PAD group (OR: 2.630, 95% CI:1.639–4.221; *P* < 0.001). No significant difference in fibrinogen and platelet count was found between the AAA + PAD group and the PAD group (*P* > 0.05).

**Table 5:** Multivariable logistics regression analysis in AAA+PAD group and PAD group.

**Risk factors**	**OR (95% CI)**	***P*-value**
Total bilirubin (umol/L)	1.189 (0.947–1.493)	0.136
Direct bilirubin (umol/L)	0.692 (0.408–1.174)	0.172
Fibrinogen (g/L)	0.793 (0.584–1.075)	0.136
D-dimer (mg/L)	2.63 (1.639–4.211)	<0.001
Platelet count, 10^9^/L	0.997 (0.992–1.002)	0.279

*CI, confidence interval; OR, odds ratio; AAA, abdominal aortic aneurysm; PAD, peripheral arterial disease.*

**Figure 3:**
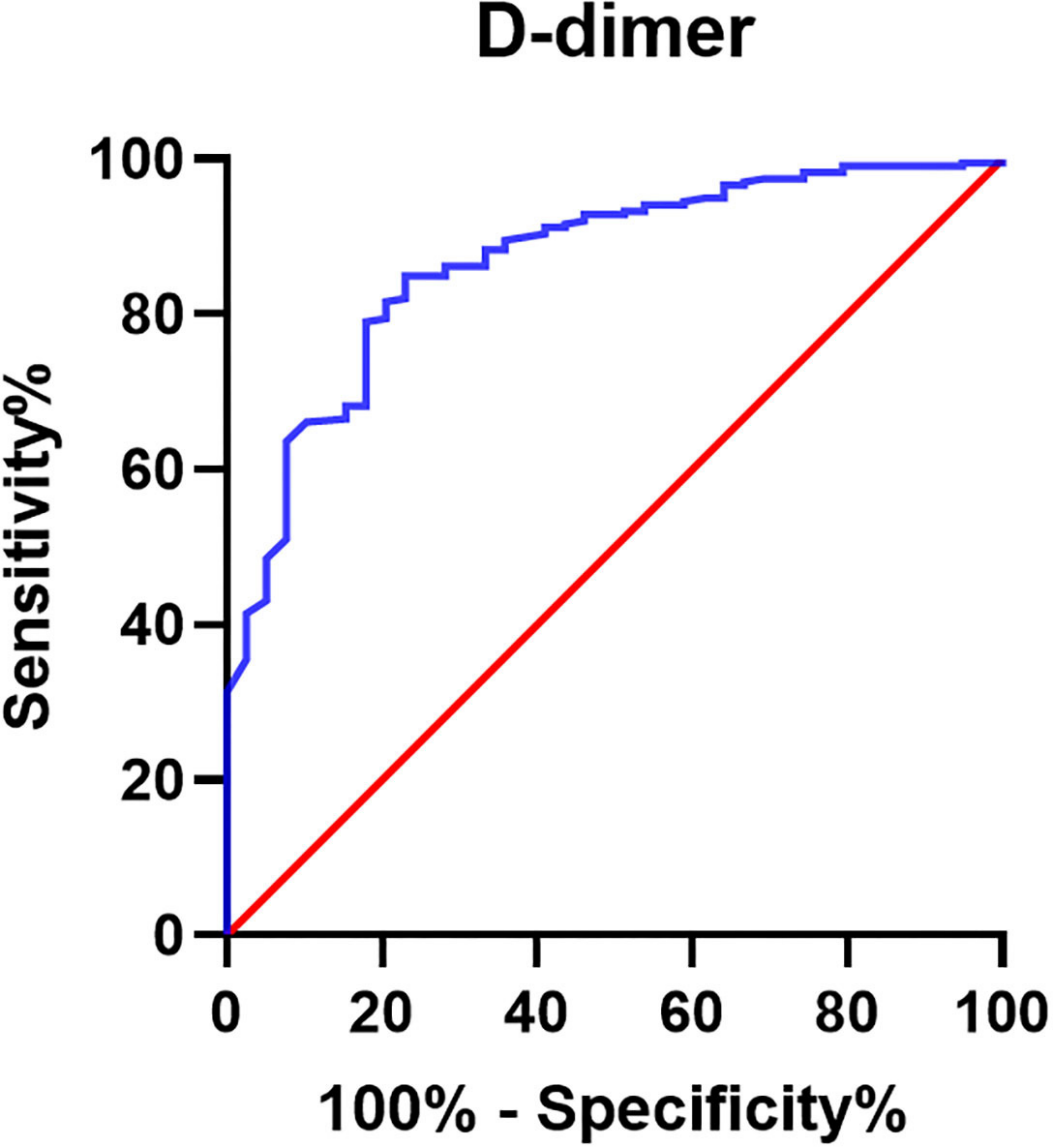
Receiver operator characteristic curve of D-dimer for prediction of AAA in PAD group. Receiver operator characteristic curve of D-dimer was drawn for the prediction of AAA in patients with PAD (n = 364). The AUC of D-dimer is 0.867.

Furthermore, the ROC curve of the D-dimer was carried out (Figure 3), and AUC was 0.867. The Youden index suggested that the best cutoff value of D-dimer was 0.675 mg/L (Table 6 with partial data provided).

**Table 6:** Diagnostic efficacy of D-dimer for AAA determined by Youden index.

**D-dimer**	**Sensitivity**	**Specificity**	**Youden index**
0.6750	0.769	0.849	0.619
0.6600	0.769	0.845	0.614
0.5050	0.821	0.791	0.611
0.5550	0.795	0.816	0.611
0.6450	0.769	0.841	0.610
0.5400	0.795	0.812	0.607
0.4950	0.821	0.782	0.603

*Youden index was determined to evaluate diagnostic efficacy of D-dimer*.

**Figure 4:**
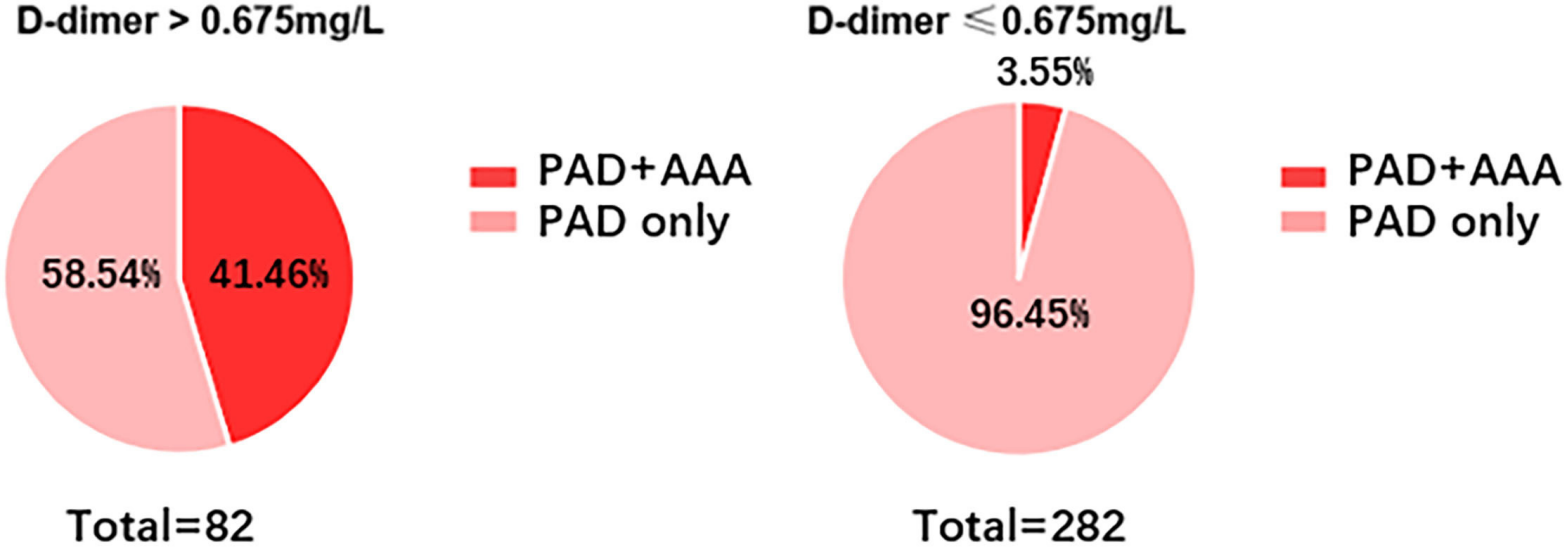
Incidence of AAA in patients with PAD with different levels of D-dimer. Incidence of AAA was determined in patients with PAD when D-dimer is ≤ 0.675 mg/L or ≥0.675 mg/L.

To better visualize the predictive efficacy of D-dimer for AAA, the 364 patients with PAD were divided into two groups based on D-dimer: D-dimer > 0.65 mg/L, and D-dimer ≤ 0.65 mg/L. As shown in Figure 4, 41.46% of patients with PAD were concomitant with AAA when D-dimer was > 0.65 mg/L, while only 3.55% of patients with PAD were concomitant with AAA when D-dimer was ≤ 0.65 mg/L.

## Discussion

In this study, we demonstrated that AAA and PAD share a group of risk factors, such as hypertension, dyslipidemia, smoking, and drinking. However, patients with AAA experienced a decreased incidence of diabetes mellitus, lowered levels of fibrinogen and platelet count, and upregulated levels of D-dimer and bilirubin. Our study further validates the diagnostic efficacy of these markers for the prediction of AAA and suggests that D-dimer is a promising biomarker with satisfying sensitivity and specificity. Patients with PAD with D-dimer of ≥0.675 indicated a highly possible presence of AAA.

Studies have revealed that hypertension, smoking, cardiovascular diseases, dyslipidemia, cerebrovascular disease, and renal insufficiency are well-defined risk factors for AAA (17–22). Researchers have also suggested that hypertensive, smoking, and dyslipidemia individuals have a higher risk of PAD (14, 23, 24). The dominant etiology of PAD and AAA is arterial atherosclerosis. Thus, it is clinically realistic that patients with PAD are concomitant with AAA (Figure 1). To date, relatively few studies have specifically focused on the potential value of a blood marker-based approach for clinicians to screen whether patients have PAD combined with AAA. Our results show that D-dimer helps predict AAA in patients with PAD in a time- and cost-effective manner over ultrasound-based screening of AAA. Coagulation status test, including D-dimer is routine, at least in China, prior to surgical treatment of PAD, thus, clinicians are less likely to ignore the dramatic increase of D-dimer in patients with PAD. The likelihood of neglecting AAA in patients with PAD is decreased significantly without the extra costs of time and money.

As is well-demonstrated, a large volume of intraluminal aortic thrombus is common in patients with AAA (25, 26), resulting from activated coagulation status inside an aneurysm. D-dimer is the degradative product of fibrin, and increased levels of D-dimer indicate the presence of coagulation procedure and secondary hyperfibrinolysis. Thus, circulating D-dimer in patients with AAA originates from fibrinolysis of intraluminal thrombus, which explains increased levels of D-dimer in patients with AAA (Table 2). Our finding is consistent with previous studies, which have confirmed the diagnostic value of D-dimer for AAA (27–30). However, control group in those studies is non-atherosclerotic population even healthy subjects. The conclusions obtained from these studies may not be applicable among athero-thrombosis diseases, for example, PAD, since AAA and PAD share common risk factors and etiologies. Our research has filled gaps in knowledge and the inclusion of patients with PAD in this study is important in distinguishing patients with AAA from PAD not only in the general population, but serum D-dimer also facilitates the diagnosis of AAA in symptomatic PAD individuals.

Currently, D-dimer is widely used as an exclusion of venous thrombo-embolism and is regarded as cost- and time-effective. As shown in Figure 4, only 3.55% of patients with PAD were diagnosed with AAA. Therefore, we can similarly take advantage of D-dimer ≤ 0.675 mg/L as an exclusion biomarker of AAA in patients with PAD.

As we know, platelet and fibrinogen are fundamental materials for coagulation, and conversion of fibrinogen to fibrin and platelet aggression are key steps in the coagulation procedure. Thus, constant coagulation will lead to the consumption of platelet and fibrinogen. This may explain the findings of the present study, that patients with AAA exerted decreased levels of platelet and fibrinogen compared with patients with PAD (Tables 2–4). These observations are consistent with previous publications (31–33).

In accordance with others, the prevalence of diabetes is decreased in patients with AAA and increased significantly in patients with PAD (Tables 1, 3). Some epidemiological studies indicate that diabetes is a protective factor for AAA and can inhibit the occurrence and progression of AAA (34, 35). The underlying mechanism may be that metformin prescription is associated with decreased AAA enlargement (36). Diabetes is also a well-recognized risk factor for PAD.

Our study has several limitations. First, the study was performed in a single-center with a limited sample size. A large scale and multicenter study is a necessity to further validate the conclusions. Second, diagnostic accuracy can be compromised when patients with PAD are experiencing atherosclerotic thrombosis due to active coagulation and fibrinolysis processes in the lower extremities.

## Conclusion

Our study has revealed differentially distributed serum markers (bilirubin, D-dimer, fibrinogen, and platelet) and the prevalence of diabetes among patients with AAA and patients with PAD. Particularly, the serum level of D-dimer is a promising biomarker for the presence of AAA in patients with PAD. A patient with PAD with a D-dimer of ≥0.675 mg/L requires further evaluation for the presence of AAA.

## Data Availability Statement

The original contributions presented in the study are included in the article/supplementary material, further inquiries can be directed to the corresponding author.

## Ethics Statement

The studies involving human participants were reviewed and approved by Ethics Committee of Xiangya Hospital, Central South University. The patients/participants provided their written informed consent to participate in this study.

## Author Contributions

WW and JH conceived and designed the study. HC, LW, SL, KW, and PY collected the data. HC, BP, JX, LW, SL, and WW analyzed the data. HC and BP wrote the manuscript. BP, KW, PY, JH, and WW provided a substantial revision of the manuscript. WW obtained funding. All authors have read and approved the final manuscript.

## Funding

This study was funded by the National Natural Science Foundation of China (No. 81873525), the Clinical Research Project of Xiangya hospital, CSU, and the project was sponsored by the Scientific Research Foundation for the Returned Overseas Chinese Scholars, State Education Ministry.

## Conflict of Interest

The authors declare that the research was conducted in the absence of any commercial or financial relationships that could be construed as a potential conflict of interest. The handling editor ZL is currently organizing a Research Topic with the WW.

## Publisher's Note

All claims expressed in this article are solely those of the authors and do not necessarily represent those of their affiliated organizations, or those of the publisher, the editors and the reviewers. Any product that may be evaluated in this article, or claim that may be made by its manufacturer, is not guaranteed or endorsed by the publisher.
